# Phosphorylation of serine residue modulates cotton Di19-1 and Di19-2 activities for responding to high salinity stress and abscisic acid signaling

**DOI:** 10.1038/srep20371

**Published:** 2016-02-01

**Authors:** Li-Xia Qin, Xiao-Ying Nie, Rong Hu, Gang Li, Wen-Liang Xu, Xue-Bao Li

**Affiliations:** 1 Hubei Key Laboratory of Genetic Regulation and Integrative Biology, School of Life Sciences, Central China Normal University, Wuhan 430079, China; 2 Institute of Cotton, Shanxi Academy of Agricultural Sciences, Yuncheng 044000, China

## Abstract

Di19 (drought-induced protein 19) family is a novel type of Cys2/His2 zinc-finger proteins. In this study, we demonstrated that cotton Di19-1 and Di19-2 (GhDi19-1/-2) proteins could be phosphorylated *in vitro* by the calcium-dependent protein kinase (CDPK). Mutation of Ser to Ala in N-terminus of GhDi19-1/-2 led to the altered subcellular localization of the two proteins, but the constitutively activated form (Ser was mutated to Asp) of GhDi19-1/-2 still showed the nuclear localization. *GhDi19-1*/*-2* overexpression transgenic *Arabidopsis* seedlings displayed the hypersensitivity to high salinity and abscisic acid (ABA). However, Ser site-mutated *GhDi19-1(S116A)* and *GhDi19-2(S114A)*, and Ser and Thr double sites-mutated *GhDi19-1(S/T-A/A)* and *GhDi19-2(S/T-A/A)* transgenic *Arabidopsis* did not show the salt- and ABA-hypersensitive phenotypes. In contrast, overexpression of Thr site-mutated *GhDi19-1(T114A)* and *GhDi19-2(T112A)* in *Arabidopsis* still resulted in salt- and ABA-hypersensitivity phenotypes, like *GhDi19-1/-2* transgenic lines. Overexpression of *GhDi19-1/-2* and their constitutively activated forms in *Atcpk11* background could recover the salt- and ABA-insensitive phenotype of the mutant. Thus, our results demonstrated that Ser phosphorylation (not Thr phosphorylation) is crucial for functionally activating GhDi19-1/-2 in response to salt stress and ABA signaling during early plant development, and GhDi19-1/-2 proteins may be downstream targets of CDPKs in ABA signal pathway.

Drought, high salinity and low temperature are the most common abiotic stresses that limit distribution of plants and affect crop productivity and quality^1^,^2^. Plant adaptation to these stresses is dependent on the activation of cascades of molecular networks involving in stress perception, signal transduction and expression of specific stress-related genes^3^,^4^. Abscisic acid (ABA) as an important phytohormone is required for plant adaptation to environmental stress by affecting some physiological processes in cells during plant growth and development^5^. Several signal molecules, such as calcium ion (Ca^2+^), reactive oxygen species (ROS) and protein kinases [including mitogen-activated protein kinase (MAPK), calcium-dependent protein kinase (CDPK), and calcium/calmodulin-dependent protein kinase (CCaMK)] play important roles in ABA signal transduction for plant response to abiotic stresses^6^,^7^,^8^,^9^,^10^. For instance, overexpression of AtCPK4 or AtCPK11 in *Arabidopsis* enhanced plant ABA- and salt-sensitivity during seed germination and seedling growth, but loss-of-function mutations of CPK4 and CPK11 resulted in plant ABA- and salt-insensitive phenotype^11^.

Transcriptional modulation is thought to be one of the most important ways for plants responding and adapting to stress conditions, and a number of genes are induced or repressed in plants under abiotic stresses^12^,^13^,^14^,^15^. Previous studies revealed that plant transcription factors (TFs) (such as bZIP, HD-ZIP, NAC, MYB, MYC and AP2/ERF) are involved in plant stress response^3^,^16^. These TFs regulate expression of the stress-related genes by specifically binding to the *cis*-elements in the target promoters^17^.

Cys2/His2 (C2H2)-type zinc-finger proteins (ZFPs), also called classical TFIII-types zinc-finger proteins, represent a large family of eukaryotic transcription factors. The C2H2-type zinc finger domain is one of the well-characterized DNA-binding motifs involved in protein-DNA, protein-RNA and protein-protein interactions in plants^18^,^19^,^20^. This domain consists of approximate 30 amino acid residues with the common sequence CX_2–4_CX_3_FX_5_LX_2_HX_3–5_H, and a zinc ion is tetrahedrally coordinated by two cysteines and two histidines in order to maintain its stability^20^. In *Arabidopsis* and rice, 176 and 189 zinc-finger proteins that contain one or more zinc-finger motifs have been identified, respectively^21^,^22^. Some zinc-finger proteins play important roles in plant response to abiotic stresses. For example, *Zat10* (STZ) and *Zat12* promote plant tolerance to drought, salinity and oxidative stresses^22^,^23^,^24^. ThZF1, a C2H2-type TF from salt cress (*Thellungiella halophila*), is involved in drought and salt stresses^25^. Similarly, several C2H2-type ZFPs are related to rice response to drought and salinity stresses^4^,^26^,^27^. Loss of function of a rice DST (drought and salt tolerance) protein leads to the enhanced plant drought- and salt-tolerance^28^.

Di19 (drought-induced 19) proteins contain two unusual and evolutionarily conserved C2H2 zinc-finger domains^29^. These proteins may share a common or closely related biological function based on the conserved C2H2 zinc finger-like motif. *Arabidopsis* Di19 family comprises of seven hydrophilic protein members. *AtDi19-1* and *AtDi19-3* are rapidly induced by dehydration, while transcript amounts of *AtDi19-2* and *AtDi19-4* are increased in response to high-salinity stress. However, most of *AtDi19* genes are not transcriptionally induced by ABA^30^. Recently, a study revealed that *Arabidopsis* Di19-1 as a transcription factor participates in plant response to drought stress by binding to the TACA(A/G)T element within the promoters of *PR1* (pathogenesis-related 1), *PR2* and *PR5* genes^31^. Subsequently, we reported that AtDi19-3 as a transcriptional activator is involved in plant response to high salinity, drought, ABA and H_2_O_2_^32^. In addition, *Arabidopsis* Di19 proteins could be phosphorylated by CPK11 and CPK3 *in vitro* in a Ca^2+^-dependent manner^30^,^33^. However, the function of most Di19 proteins in plants remains unknown as yet. Our previous study revealed that two cotton Di19 proteins (GhDi19-1 and GhDi19-2) are involved in plant response to salt stress and ABA signaling. Overexpression of *GhDi19-1* and *GhDi19-2* in *Arabidopsis* enhanced plant sensitivity to high salinity and exogenous ABA^34^. In this study, we further demonstrated that the serine (Ser) site in N-terminus of the GhDi19-1/-2 proteins is crucial for functionally activating GhDi19-1/-2 in response to salt stress and ABA signaling during early seedling development. The calcium-dependent protein kinase (CDPK) is able to phosphorylate these Di19 proteins at Ser116 of GhDi19-1 and at Ser114 of GhDi19-2.

## Results

### GhDi19-1/-2 is phosphorylated by calcium-dependent protein kinase (CDPK) in a Ca^2+^-dependent manner

**Figure 1:**
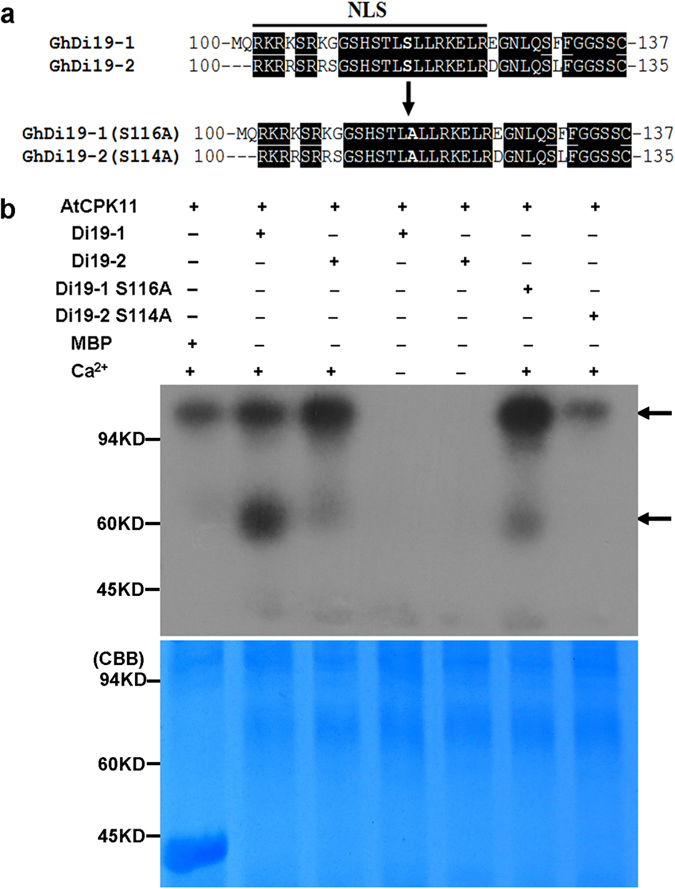
*In* vitro assay of GhDi19-1 and GhDi19-2 phosphorylation. (**a**) The map of mutation of specific amino acids in the phosphorylation of GhDi19-1 and GhDi19-2. NLS, nuclear localization signal domain; S, serine (Ser); A, alanine (Ala). The arrowhead indicated the mutated amino acids. (**b**) AtCPK11 phosphorylates GhDi19-1 and GhDi19-2 *in vitro*. Top: Autoradiograph showing AtCPK11 autophosphorylation and GhDi19-1/-2 phosphorylation; Bottom: the same SDS-PAGE gel with Coomasie Brilliant Blue (CBB)–stained AtCPK11 and GhDi19-1/-2 proteins. GhDi19-1 and GhDi19-2 proteins were purified as a MBP-fusion and incubated with AtCPK11-MBP. A control reaction with the MBP protein was also performed. The upper arrowhead indicated autophosphorylation of AtCPK11-MBP, while the lower arrowhead indicated phosphorylation of GhDi19-1-MBP and GhDi19-2-MBP.

Sequence analysis showed that both GhDi19-1 and GhDi19-2 contain Ser/Thr kinase phosphorylation sites in the conserved nuclear localization signal region next to the two zinc finger domains (GhDi19-1: Thr114 and Ser116; GhDi19-2: Thr112 and Ser114), and the predicted Ser phosphorylation is potentially stronger than the Thr phosphorylation (http://myhits.isb-sib.ch/cgi-bin/motif_scan). Then, we employed an *in vitro* kinase assay to determine whether GhDi19-1 and GhDi19-2 are phosphorylated by a kinase (such as AtCPK11). As shown in Fig. 1a, the S_116_ of GhDi19-1 was mutated to A_116_ [GhDi19-1(S116A)], and the S_114_ of GhDi19-2 was mutated to A_114_ [GhDi19-2(S114A)]. The *in vitro* kinase assay revealed that the recombinant AtCPK11 phosphorylated GhDi19-1 and GhDi19-2 in the presence of Ca^2+^. Additionally, weaker phosphorylation of GhDi19-1(S116A) was detected in the presence of Ca^2+^, and no phosphorylation of GhDi19-2(S114A) was found with or without Ca^2+^ (Fig. 1b). The above results indicated that GhDi19-1/-2 is phosphorylated *in vitro* by a kinase (such as AtCPK11) in a Ca^2+^-dependent manner, and the 116th Ser of GhDi19-1 or the 114th Ser of GhDi19-2 is required for the phosphorylation.

### Ser site is crucial for normal subcellular localization of GhDi19-1 and GhDi19-2 proteins

**Figure 2:**
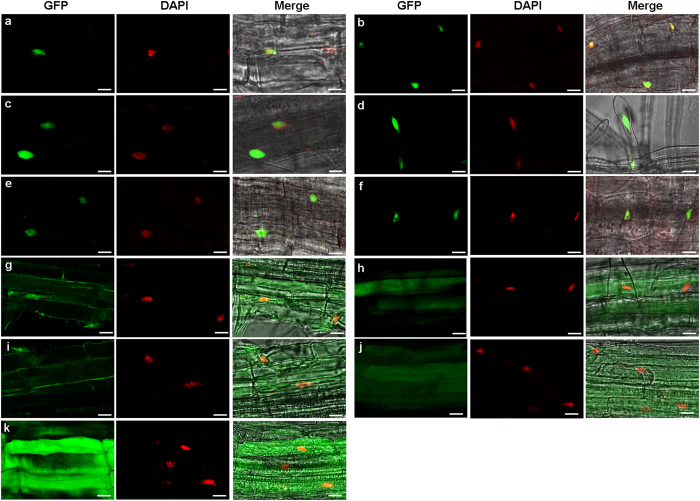
Subcellular localization of GhDi19-1/-2 and their mutated proteins in cells. (**a–f**) GFP fluorescence signals were mainly detected in nuclei of the transgenic *Arabidopsis* root cells harboring the GhDi19-1:eGFP, GhDi19-2:eGFP, GhDi19-1(S116D):eGFP, GhDi19-2(S114D):eGFP, GhDi19-1(T114A):eGFP and GhDi19-2(T112A):eGFP, respectively. (**a,b**) Normal **s**ubcellular localization of GhDi19-1 (**a**) and GhDi19-2 (**b**). (**c,d**) Subcellular localization of the Ser site-mutated (constitutively activated) GhDi19-1(S116D) (**c**) and GhDi19-2(S114D) (**d**). (**e,f**) Subcellular localization of the Thr site-mutated GhDi19-1(T114A) (**e**) and GhDi19-2 (T112A) (**f**). (**g–j**) The altered **s**ubcellular localization of the Ser site-mutated GhDi19-1(S116A) **(g)**, GhDi19-2(S114A) **(h)**, the Ser and Thr double sites-mutated GhDi19-1(S/T-A/A) **(i)** and GhDi19-2(S/T-A/A) **(j)**. GFP fluorescence signals were detected in cytoplasm, cytomembrane and nuclei of the transgenic *Arabidopsis* root cells. (**k**) pBI121-eGFP construct was used as positive control. (Left) Confocal microsopy of GFP fluorescence. (Midst) Nuclear DAPI staining of the same cell on the left. (Right) Left and midst superimposed over the bright-field image. Bars = 10 μm. S116D, the S_116_ of GhDi19-1 was mutated to D_116_; S114D, the S_114_ of GhDi19-2 was mutated to D_114_; T114A, the T_114_ of GhDi19-1 was mutated to A_114_; T112A, the T_112_ of GhDi19-2 was mutated to A_112_; S116A, the S_116_ of GhDi19-1 was mutated to A_116_; S114A, the S_114_ of GhDi19-2 was mutated to A_114_; S/T-A/A, the S_116_ and T_114_ of GhDi19-1 were mutated to A_116_ and A_114_, and the S_114_ and T_112_ of GhDi19-2 were mutated to A_114_ and A_112_, respectively. S, serine (Ser); A, alanine (Ala); T, threonine (Thr); D, aspartic acid (Asp).

Bioinformatics analysis predicted that both GhDi19-1 and GhDi19-2 contain a conserved nuclear localization signal (NLS) in its sequence. To confirm nuclear localization of the two GhDi19 proteins, the coding regions of the two genes were fused with an enhanced GFP (eGFP) reporter gene under the control of a cauliflower mosaic virus (CaMV) 35S promoter, respectively, and expressed constitutively in *Arabidopsis*. To determine whether Ser site and/or Thr site play an essential role in subcellular localization of GhDi19-1 and GhDi19-2 proteins, the S_116_ of GhDi19-1 was mutated to A_116_ [GhDi19-1(S116A)], the S_114_ of GhDi19-2 was mutated to A_114_ [GhDi19-2(S114A)], the T_114_ of GhDi19-1 was mutated to A_114_ [GhDi19-1(T114A)], the T_112_ of GhDi19-2 was mutated to A_112_ [GhDi19-2(T112A)], both the T_114_ and S_116_ of GhDi19-1 were mutated to A [GhDi19-1(S/T-A/A)], and both the T_112_ and S_114_ of GhDi19-2 were mutated to A [GhDi19-2(S/T-A/A)]. Furthermore, S_116_ was mutated to D_116_ in GhDi19-1 and S_114_ was mutated to D_114_ in GhDi19-2 for generating the constitutively activated GhDi19-1 and GhDi19-2 [named GhDi19-1(S116D) and GhDi19-2(S114D), respectively]. The coding sequences of the above mutated *GhDi19s* were also fused with an enhanced GFP (eGFP) reporter gene under the control of a cauliflower mosaic virus (CaMV) 35S promoter, respectively, and expressed constitutively in *Arabidopsis*. As shown in Fig. 2a,b, the GhDi19-1:eGFP and GhDi19-2:eGFP fusion proteins were mainly accumulated in the nuclei of root cells of the transgenic seedlings, demonstrating that GhDi19-1 and GhDi19-2 are two nuclear-localized proteins. Likewise, strong GFP fluorescence signals were detected in the nuclei of the transgenic root cells harboring the GhDi19-1(S116D):eGFP (Fig. 2c), GhDi19-2(S114D):eGFP (Fig. 2d), GhDi19-1(T114A):eGFP (Fig. 2e), and GhDi19-2(T112A):eGFP (Fig. 2f), respectively. On the contrary, GFP fluorescence was found in cytoplasm, cytomembrane and nuclei of root cells of the transgenic plants expressing Ser site-mutated GhDi19-1(S116A) (Fig. 2g) and GhDi19-2(S114A) (Fig. 2h), and Ser/Thr double sites-mutated GhDi19-1(S/T-A/A) (Fig. 2i) and GhDi19-2(S/T-A/A) (Fig. 2j), indicating that subcellular localization of both GhDi19-1 and GhDi19-2 proteins was altered as the Ser site (but not Thr site) was mutated to Ala in these proteins, while GFP fluorescence signals were observed in whole cells of the transgenic seedlings harboring only GFP vector (Fig. 2k). The above data implied that the Ser site (but not Thr site) is essential for normal subcellular localization of GhDi19-1 and GhDi19-2 proteins.

### Ser site is crucial for activating GhDi19-1 and GhDi19-2 proteins in response to salt stress and ABA signaling

**Figure 3:**
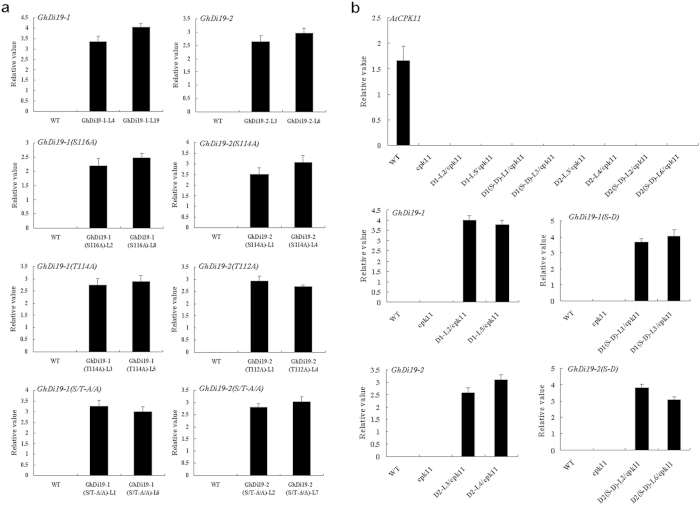
Quantitative RT-PCR analysis of expression of the related exogenous *GhDi19-1/-2* genes in transgenic *Arabidopsis*. (**a**) Quantitative RT-PCR analysis of expression of the *GhDi19-1/-2* and their mutant genes in wild type, and the *GhDi19-1/-2* and their site-mutated transgenic lines. 10-day-old seedlings were used for RNA extraction. WT, wild type; Di19-1-L4 and -L19, the *GhDi19-1* transgenic line 4 and 19; GhDi19-1(S116A)-L2 and -L8, the site-mutated *GhDi19-1(S116A)* transgenic line 2 and 8 (S116A, the S_116_ of GhDi19-1 was mutated to A_116_). GhDi19-1(T114A)-L3 and -L5, the site-mutated *GhDi19-1(T114A)* transgenic line 3 and 5 (T114A, the T_114_ of GhDi19-1 was mutated to A_114_). GhDi19-1(S/T-A/A)-L1 and -L6, the double sites-mutated *GhDi19-1(S/T-A/A)* transgenic line 1 and 6 (S/T-A/A, the S_116_ and T_114_ of GhDi19-1 were mutated to A_116_ and A_114_, respectively). GhDi19-2-L3 and -L6, the *GhDi19-2* transgenic line 3 and 6; GhDi19-2(S114A)-L1 and -L4, the site-mutated *GhDi19-2(S114A)* transgenic line 1 and 4 (S114A, the S_114_ of GhDi19-2 was mutated to A_114_); GhDi19-2(T112A)-L1 and -L4, the site-mutated *GhDi19-2(T112A)* transgenic line 1 and 4 (T112A, the T_112_ of GhDi19-2 was mutated to A_112_); GhDi19-2(S/T-A/A)-L2 and -L7, the double sites-mutated *GhDi19-2(S/T-A/A)* transgenic line 2 and 7 (S/T-A/A, the S_114_ and T_112_ of GhDi19-2 were mutated to A_114_ and A_112_, respectively). (**b**) Quantitative RT-PCR analysis of expression of the *GhDi19-1/-2* and their mutant genes in wild type, *Atcpk11* mutant, and GhDi19-1/2- and their mutated transgenic lines in *Atcpk11* mutant. 10-day-old seedlings were used for RNA extraction. WT, wild type; cpk11, *Atcpk11*-2 mutant; *D1-L2/cpk11* and *D1-L5/cpk11*, the *GhDi19-1* overexpression transgenic line 2 and 5 in *Atcpk11*-2 mutant; *D1(S-D)-L1/cpk11* and *D1(S-D)-L3/cpk11*, the site-mutated *GhDi19-1(S-D)* overexpression transgenic line 1 and 3 in *Atcpk11*-2 mutant; *D2-L3/cpk11* and *D2-L4/cpk11*, the *GhDi19-2* overexpression transgenic line 3 and 4 in *Atcpk11*-2 mutant; *D2(S-D)-L2/cpk11* and *D2(S-D)-L6/cpk11*, the site-mutated *GhDi19-2(S-D)* overexpression transgenic line 2 and 6 in *Atcpk11*-2 mutant (S-D, the S_116_ of GhDi19-1 was mutated to D_116_ and the S_114_ of GhDi19-2 was mutated to D_114_). The assays were repeated three times along with three independent repetitions of the biological experiments. S, serine (Ser); A, alanine (Ala); T, threonine (Thr); D, aspartic acid (Asp).

Our previous study indicated that overexpression of the *GhDi19-1* and *GhDi19-2* genes in *Arabidopsis* enhances plant sensitivity to salt stress in ABA-dependent manner^34^. To further investigate the mechanism of GhDi19-1 and GhDi19-2 involving in response to salt stress and ABA signaling, the S_116_ of GhDi19-1 was mutated to A_116_, and the S_114_ of GhDi19-2 was mutated to A_114_. The coding sequences of *GhDi19-1(S116A)* and *GhDi19-2(S114A)* were inserted into plant expression vector pBI121 driven by CaMV 35S promoter, and introduced into *Arabidopsis*, respectively. Homozygotes of T3 generation of *GhDi19-1* overexpression transgenic lines (L4 and L19), *GhDi19-1(S116A)* overexpression transgenic lines (L2 and L8), *GhDi19-2* overexpression transgenic lines (L3 and L6) and *GhDi19-2(S114A)* overexpression transgenic lines (L1 and L4) were analyzed by PCR to ensure the presence of the respective transgene and by quantitative RT-PCR to confirm transgene expression (Fig. 3a).

**Figure 4:**
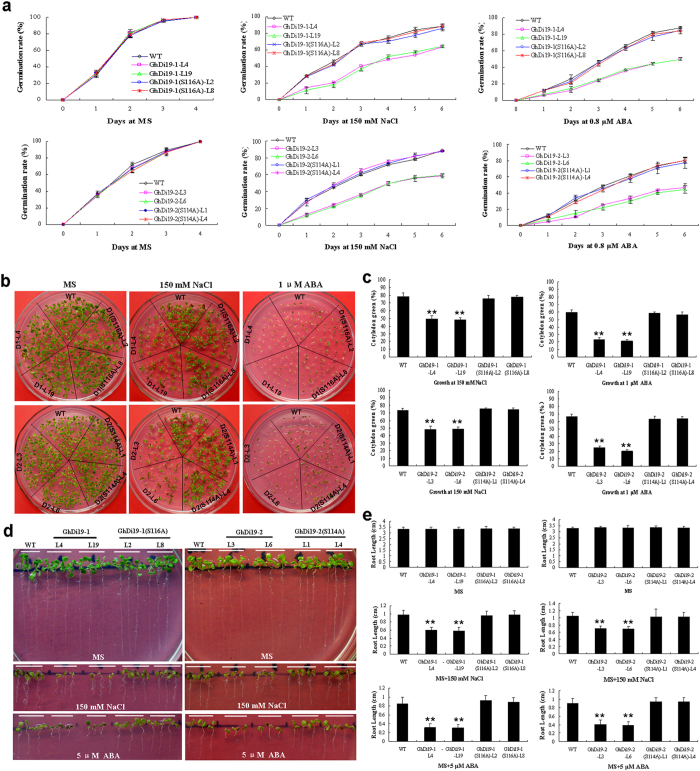
Effect of serine site-mutation on GhDi19-1 and GhDi19-2 responding to salt and abscisic acid (ABA) during seed germination and early seedling development. (**a**) Statistical analysis of seed germination rate. Seeds of the transgenic lines and wild type were germinated on MS medium without (control) or with 150 mM NaCl and 0.8 μM ABA, respectively. (**b**) Growth status of the transgenic seedlings and wild type grown on MS medium without or with 150 mM NaCl and 1 μM ABA for 10 days, respectively. (**c**) Statistical analysis of cotyledon expansion and greening of seedlings grown on MS medium with 150 mM NaCl and 1 μM ABA for 10 days, respectively. (**d**) Phenotypic analysis of GhDi19-1/-2(S-A) transgenic *Arabidopsis* under NaCl and ABA treatments. 10-day-old seedlings of wild type, GhDi19-1/-2 and GhDi19-1/-2(S-A)-overexpression transgenic lines growing on MS medium with or without 150 mM NaCl and 5 μM ABA, respectively. (**e**) Statistical analysis of root length of 10-day-old seedlings of wild type and GhDi19-1/2 and GhDi19-1/2(S-A) transgenic lines growing on MS medium without (control) or with 150 mM NaCl and 5 μM ABA, respectively. Mean values and standard errors (bar) were shown from three independent experiments (n > 60 seedlings per each line). Asterisk represents that there was (very) significant difference between the transgenic lines and wild type in independent *t*-tests (one asterisk: P value<0.05; two asterisks: P value<0.01). WT, wild type; GhDi19-1-L4 (D1-L4) and -L19 (D1-L19), the *GhDi19-1* transgenic line 4 and 19; GhDi19-1(S116A)-L2 [D1(S116A)-L2] and -L8 [D1(S116A)-L8], the site-mutated *GhDi19-1(S116A)* transgenic line 2 and 8 (S116A, the S_116_ of GhDi19-1 was mutated to A_116_); Di19-2-L3 (D2-L3) and -L6 (D2-L6), the *GhDi19-2* transgenic line 3 and 6. GhDi19-2(S114A)-L1 [D2(S114A)-L1] and -L4 [D2(S114A)-L4], the site-mutated *GhDi19-2(S114A)* transgenic line 1 and 4 (S114A, the S_114_ of GhDi19-2 was mutated to A_114_). S, serine (Ser); A, alanine (Ala).

Seeds of *GhDi19-1*, *GhDi19-1(S116A)*, *GhDi19-2* and *GhDi19-2(S114A)* overexpression transgenic lines and wild type were sowed on MS medium with or without NaCl and ABA, respectively. On MS medium, all the seeds from wild type and *GhDi19-1/-2* variants were able to fully germinate (≤ 4 days) after sowing, and there was no significant difference in seed germination rate between each other. In the presence of 150 mM NaCl, however, the *GhDi19-1* and *GhDi19-2* transgenic seeds germinated much later than those of wild type. After 4 day, approximately 75% of wild type seeds germinated, but only about 35%–40% of *GhDi19-1* and *GhDi19-2* transgenic seeds germinated. After 6 days, germination rate of wild type seeds reached to about 90%, but both *GhDi19-1* and *GhDi19-2* transgenic seeds reached to only approximately 60%. In contrast, seed germination rates of *GhDi19-1(S116A)* and *GhDi19-2(S114A)* transgenic lines were similar to those of wild type with NaCl treatment. On MS medium supplemented with 0.8 μM ABA, seed germination rate of both *GhDi19-1* and *GhDi19-2* transgenic lines was significantly lower than that of wild-type. About 60% of wild type seeds, but only 40% of *GhDi19-1* and 30% of *GhDi19-2* overexpression transgenic seeds germinated after 4 days. On the contrary, seed germination rate of *GhDi19-1(S116A)* and *GhDi19-2(S114A)* overexpression transgenic lines was as same as those of wild type under ABA treatment (Fig. 4a).

To assess effects of Ser site on GhDi19-1 and GhDi19-2 involving in response to salt and ABA during early seedling development, we used two approaches in the experiments. One way is that seeds were directly planted on MS medium containing 150 mM NaCl or 1 μM ABA for determining growth status of the seedlings after germination (Fig. 4b). Another is that seeds were germinated on MS medium for 48 h after stratification and then transferred onto MS medium containing 150 mM NaCl and 5 μM ABA in the vertical position (Fig. 4d). The results obtained with these two approaches were similar. There was no significant difference among the *GhDi19-1/-2* and their site-mutated transgenic lines and wild type when seedlings grew on MS medium. However, the *GhDi19-1* and *GhDi19-2* overexpression seedlings were more sensitive to salt and ABA than wild type, but growth status of *GhDi19-1(S116A)* and *GhDi19-2(S114A)* transgenic seedlings was almost as same as those of wild type (Fig. 4b). The cotyledon greening rates of *GhDi19-1* overexpression lines (L4 and L19) and *GhDi19-2* overexpression lines (L3 and L6) were significantly lower than those of wild type under NaCl and ABA treatments, while cotyledon greening rates of *GhDi19-1(S116A)* and *GhDi19-2(S114A)* transgenic lines (L2 and L8, L1 and L4, respectively) were similar to those of wild type (Fig. 4c).

When the seedlings were transferred onto MS plates in the vertical position without any stress treatments, root growth of all *GhDi19-1/-2* transgenic seedlings and their site-variants was almost as same as those of wild type (Fig. 4d). However, when the seedlings were transferred onto MS medium supplemented with 150 mM NaCl or 5 μM ABA for several days, primary root growth of the GhDi19-1 and GhDi19-2 overexpression transgenic seedlings was significantly inhibited much more than that of wild type, but growth of *GhDi19-1(S116A) and GhDi19-2(S114A)* transgenic roots was similar to that of wild type (Fig. 4d). Under NaCl and ABA treatments, roots of GhDi19-1 and GhDi19-2 overexpression seedlings were shorter than those of wild type, whereas there was little significant difference in primary root length between *GhDi19-1(S116A)* and *GhDi19-2(S114A)* transgenic lines and wild type (Fig. 4e). In addition, we also introduced the “empty vector” (pBI121-eGFP) into *Arabidopsis* as a negative control in the above experiments, and found that the phenotypes of the transgenic plants harboring the empty vector were similar to those of wild type. Collectively, these data suggested that overexpression of *GhDi19-1* and *GhDi19-2* in *Arabidopsis* enhances plant sensitivity to salt stress in ABA-dependent manner, and the Ser site is essential for GhDi19-1 and GhDi19-2 proteins involving in plant response to salt and ABA during seed germination and early seedling development.

### Thr site is inessential for GhDi19-1 and GhDi19-2 in response to salt stress and ABA signaling

To understand whether Thr phosphorylation site also play an important role in GhDi19-1 and GhDi19-2 involving in response to salt and ABA signaling, the T_114_ of GhDi19-1 was mutated to A_114_, the T_112_ of GhDi19-2 was mutated to A_112_, both T_114_ and S_116_ of GhDi19-1 were mutated to A, and both T_112_ and S_114_ of GhDi19-2 were mutated to A. The coding sequences of *GhDi19-1(T114A), GhDi19-1(S/T-A/A), GhDi19-2(T112A)* and *GhDi19-2(S/T-A/A)* were inserted into pBI121 vector, and introduced into *Arabidopsis*, respectively. Homozygotes of T3 generation of the respective overexpression transgenic lines were analyzed by PCR to ensure confirm the presence of the respective transgene and by quantitative RT-PCR to confirm that the transgene was expressed (Fig. 3a). Two transgenic lines (L3 and L5) with higher *GhDi19-1(T114A)* expression, two lines (L1 and L6) with higher *GhDi19-1(S/T-A/A)* expression, two lines (L1 and L4) with higher *GhDi19-2(T112A)* expression and two lines (L2 and L7) with higher *GhDi19-2(S/T-A/A)* expression were selected for analyzing their phenotypes under different stresses.

**Figure 5:**
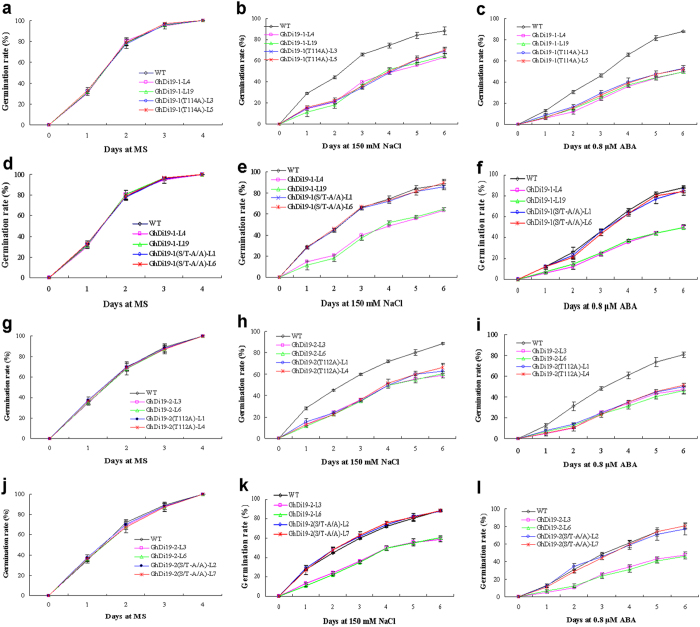
Effect of threonine site-mutation on GhDi19-1 and GhDi19-2 responding to salt and abscisic acid (ABA) during seed germination. Seeds of the transgenic lines and wild type were germinated on MS medium (**a,d,g,j**), MS medium with 150 mM NaCl (**b,e,h,k**) and MS medium with 0.8 μM ABA (**c,f,i,l**), respectively. Statistical analysis of seed germination rate was determined. Mean values and standard errors (bar) were shown from three independent experiments (n > 200 seeds per each line). WT, wild type; Di19-1-L4 and -L19, the *GhDi19-1* transgenic line 4 and 19; GhDi19-1(T114A)-L3 and -L5, the site-mutated *GhDi19-1(T114A)* transgenic line 3 and 5; GhDi19-1(S/T-A/A)-L1 and -L6, the double sites-mutated *GhDi19-1(S/T-A/A)* transgenic line 1 and 6 (T114A, the T_114_ of GhDi19-1 was mutated to A_114_; S/T-A/A, the S_116_ and T_114_ of GhDi19-1 were mutated to A_116_ and A_114_). Di19-2-L3 and -L6, the *GhDi19-2* transgenic line 3 and 6; GhDi19-2(T112A)-L1 and -L4, the site-mutated *GhDi19-1(T112A)* transgenic line 1 and 4; GhDi19-1(S/T-A/A)-L2 and -L7, the double sites-mutated *GhDi19-2(S/T-A/A)* transgenic line 2 and 7 (T112A, the T_112_ of GhDi19-2 was mutated to A_112_; S/T-A/A, the S_114_ and T_112_ of GhDi19-2 were mutated to A_114_ and A_112_). The assays were repeated three times along with three independent repetitions of the biological experiments. S, serine (Ser) ; T, threonine (Thr); A, alanine (Ala).

When seeds sowed on MS medium, there was no significant difference in seed germination between all the transgenic lines and wild type under normal conditions (Fig. 5a,d,g, j). When sowing on MS medium containing 150 mM NaCl or 0.8 μM ABA, however, the *GhDi19-1(T114A)* and *GhDi19-2(T112A)* transgenic seeds germinated much later than those of wild type, and showed salt- and ABA-sensitivity. In the presence of 150 mM NaCl, only about 35%–40% of the *GhDi19-1(T114A)* and *GhDi19-2(T112A)* transgenic seeds germinated, while approximately 65% of wild type germinated after 3 days. And germination rate of the transgenic seeds only reached to 60%, while wild type was about 80% after five days of germination (Fig. 5b,h). In addition, seed germination rate of the *GhDi19-1(T114A)* and *GhDi19-2(T112A)* transgenic lines was reduced much more than that of wild type under ABA treatment. When treated with 0.8 μM ABA, the seed germination rates of *GhDi19-1*, *GhDi19-2*, *GhDi19-1(T114A)* and *GhDi19-2(T112A)* transgenic lines were decreased to approximately 40%, whereas wild type retained 75% seed germination rate after 4 days (Fig. 5c,i). On the other hand, there was no significant difference in seed germination rate between *GhDi19-1(S/T-A/A)* and *GhDi19-2(S/T-A/A)* transgenic lines and wild type under NaCl and ABA treatments (Fig. 5e,k,f,l).

**Figure 6:**
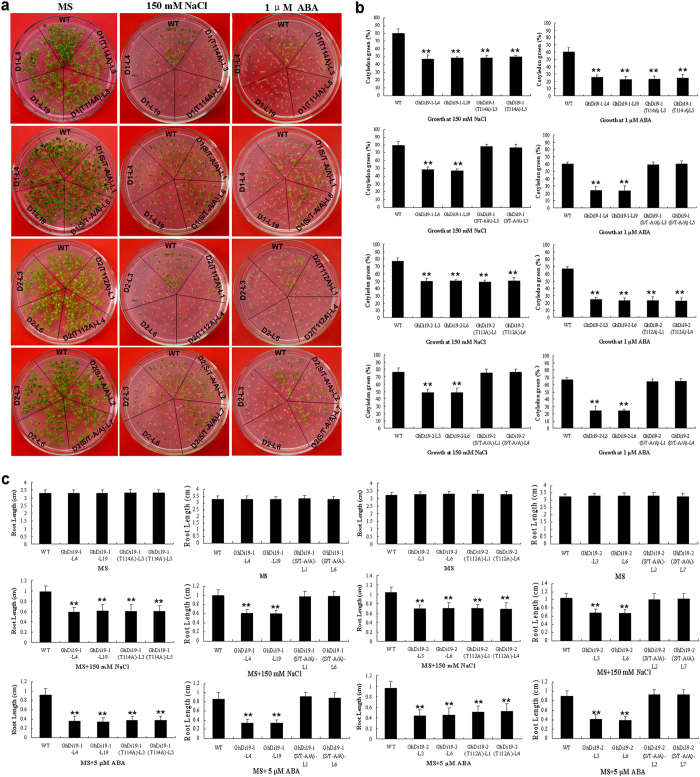
Effect of threonine site-mutation on GhDi19-1 and GhDi19-2 responding to salt and abscisic acid (ABA) during early seedling development. (**a**) Growth status of wild type, and the *GhDi19-1/-2*, *GhDi19-1/-2(T-A)* and *GhDi19-1/-2(S/T-A/A)* overexpression seedlings growing on MS medium with or without 150 mM NaCl and 1 μM ABA, respectively. (**b**) Statistical analysis of cotyledon expansion and greening of wild type, and the *GhDi19-1/-2*, *GhDi19-1/-2(T-A)* and *GhDi19-1/-2(S/T-A/A)* overexpression seedlings growing on MS medium with 150 mM NaCl and 1 μM ABA, respectively. (**c**) Statistical analysis of root length of 10-day-old seedlings of wild type, and the *GhDi19-1/-2*, *GhDi19-1/-2(T-A)* and *GhDi19-1/-2(S/T-A/A)* overexpression lines growing on MS medium without (control) or with 150 mM NaCl and 5 μM ABA. Mean values and standard errors (bar) were shown from three independent experiments (n > 60 seedlings per each line). Asterisk represents that there was (very) significant difference between the transgenic lines and wild type in independent *t*-tests (one asterisk: P value<0.05; two asterisks: P value<0.01). WT, wild type; GhDi19-1-L4 (D1-L4) and -L19 (D1-L19), the *GhDi19-1* transgenic line 4 and 19; GhDi19-1(T114A)-L3 and -L5 [D1(T114A)-L3 and -L5], the site-mutated *GhDi19-1(T114A)* transgenic line 3 and 5; GhDi19-1(S/T-A/A)-L1 and -L6 [D1(S/T-A/A)-L1 and -L6], the double sites-mutated *GhDi19-1(S/T-A/A)* transgenic line 1 and 6 (T114A indicated the T_114_ of GhDi19-1 was mutated to A_114_, and S/T-A/A indicated both S_116_ and T_114_ of GhDi19-1 were mutated to A_116_ and A_114_). GhDi19-2-L3 (D1-L3) and -L6 (D1-L6), the *GhDi19-2* transgenic line 3 and 6; GhDi19-2(T112A)-L1 and -L4 [D2(T112A)-L1 and -L4], the site-mutated *GhDi19-2(T112A)* transgenic line 1 and 4; GhDi19-2(S/T-A/A)-L2 and -L7 [D2(S/T-A/A)-L2 and -L7], the double sites-mutated *GhDi19-2(S/T-A/A)* transgenic line 2 and 7 (T112A indicated the T_112_ of GhDi19-2 was mutated to A_112_, and S/T-A/A indicated both S_114_ and T_112_ of GhDi19-2 were mutated to A_114_ and A_112_). S, serine (Ser); T, threonine (Thr); A, alanine (Ala).

We also calculated cotyledon greening of the transgenic seedlings and wild type under salt stress. As shown in Fig. 6a, both *GhDi19-1(T114A)* and *GhDi19-2(T112A)* transgenic seedlings growing on MS containing 150 mM NaCl were more sensitive than wild type. Cotyledon greening of *GhDi19-1(T114A)* and *GhDi19-2(T112A)* transgenic seedlings was drastically inhibited by NaCl treatment, compared with that of wild type. Similarly, cotyledon greening of *GhDi19-1(T114A)* and *GhDi19-2(T112A)* transgenic plants was also inhibited more than that of wild type. In the presence of 1 μM ABA, approximately 20% and 25% of the *GhDi19-1(T114A)* and *GhDi19-2(T112A)* transgenic seedlings with expanded and turned-green cotyledons was observed, respectively, while wild type reached to 60% cotyledon greening rate. In contrast, *GhDi19-1(S/T-A/A)* and *GhDi19-2(S/T-A/A)* transgenic plants showed the similar phenotype to wild-type under NaCl and ABA treatments (Fig. 6b).

Additionally, seeds of all the transgenic *Arabidopsis* lines and wild type germinated on MS medium for 48 h, and then were transferred onto NaCl- and ABA-containing MS medium for investigating growth of the seedlings responding to high salinity and ABA (Fig. 6c). When transferred onto MS plates, root growth of all the transgenic seedlings was almost as same as that of wild type. However, when the seedlings were transferred onto MS medium supplemented with 150 mM NaCl or 5 μM ABA for several days, primary roots growth of the *GhDi19-1(T114A)* and *GhDi19-2(T112A)* transgenic seedlings was more significantly inhibited than that of wild type by NaCl or ABA. The roots of *GhDi19-1(T114A)* and *GhDi19-2(T112A)* transgenic seedlings were shorter than those of wild type under NaCl and ABA treatments. However, there was no significant difference in root length between *GhDi19-1(S/T-A/A)* and *GhDi19-2(S/T-A/A)* transgenic lines and wild type under NaCl and ABA treatments (Fig. 6c). These results demonstrated that overexpression of the mutated *GhDi19-1(T114A)* and *GhDi19-2(T112A)* genes in *Arabidopsis* still resulted in the transgenic plants salt- and ABA-hypersensitivity, like *GhDi19-1/-2 *transgenic lines, suggesting that the Thr site is inessential for GhDi19-1 and GhDi19-2 proteins involving in response to salt stress and ABA signaling during seed germination and early seedling development.

### Phosphorylation of Ser site by calcium-dependent protein kinase (CDPK) is important for functionally activating GhDi19-1/-2 in response to salt stress and ABA signaling

**Figure 7:**
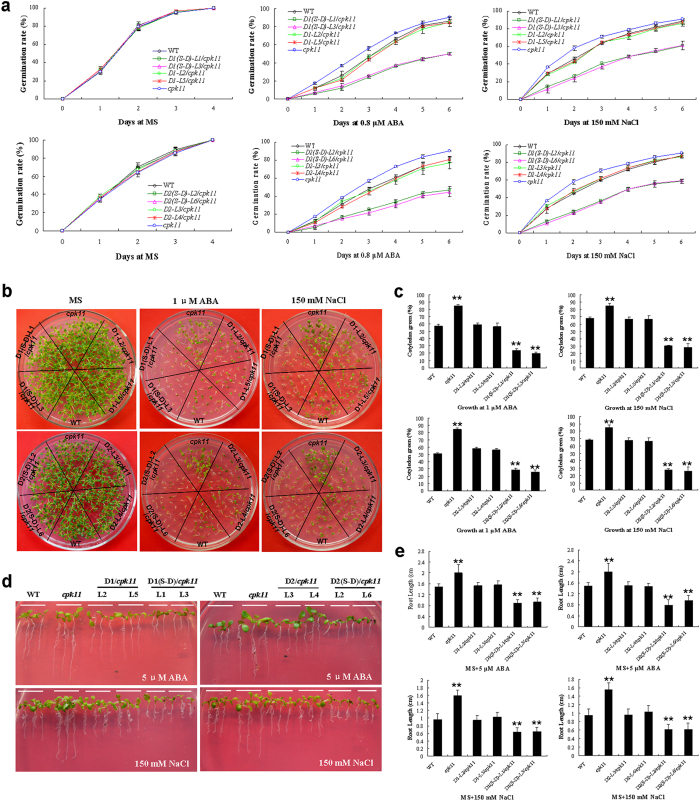
Phenotypic assay of *GhDi19-1/-2* and their activated form *GhDi19-1/-2* (S-D) transgenic seedlings in *Atcpk11* mutant under salt and abscisic acid (ABA) treatments. (**a**) Statistical analysis of seed germination rate. Seeds of wild type, *Atcpk11* mutant, and GhDi19-1/-2 or GhDi19-1/-2(S-D) overexpression transgenic lines in *Atcpk11* mutant were germinated on MS medium with or without 150 mM NaCl and 0.8 μM ABA, respectively. (**b**) Growth status of seedlings growing on MS medium with or without 1 μM ABA and 150 mM NaCl for 10 days, respectively. (**c**) Statistical analysis of cotyledon expansion and greening of seedlings growing on MS medium with 1 μM ABA and 150 mM NaCl for 10 days, respectively. (**d**) Phenotypes of seedlings under NaCl and ABA treatments. After germination on MS medium for 48 h, the wild type, *Atcpk11* mutant, and *GhDi19-1/-2* or *GhDi19-1/-2(S-D)* overexpression transgenic seedlings in *Atcpk11* mutant were transferred and grew for 10 days on MS medium without or with 150 mM NaCl and 5 μM ABA, respectively, in the vertical position. (**e**) Statistical analysis of root length of 10-day-old seedlings growing on MS medium with 5 μM ABA and 150 mM NaCl. Mean values and standard errors (bar) were shown from three independent experiments (n > 60 seedlings per each line). Asterisk represents that there was (very) significant difference between the transgenic lines and wild type in independent *t*-tests (one asterisk: P value<0.05; two asterisks: P value<0.01). WT, wild type; *cpk11*, the *Atcpk11*-2 mutant; *D1-L2/cpk11* and *D1-L5/cpk11*, the *GhDi19-1* overexpression transgenic line 2 and 5 in *Atcpk11*-2 mutant; *D1(S-D)-L1/cpk11* and *D1(S-D)-L3/cpk11*, the *GhDi19-1(S-D)* overexpression transgenic line 1 and 3 in *Atcpk11*-2 mutant; *D2-L3/cpk11* and *D2-L4/cpk11*, the *GhDi19-2* overexpression transgenic line 3 and 4 in *Atcpk11*-2 mutant; *D2(S-D)-L2/cpk11* and *D2(S-D)-L6/cpk11*, the *GhDi19-2(S-D)* overexpression transgenic line 2 and 6 in *Atcpk11*-2 mutant. S-D, the S_116_ of GhDi19-1 was mutated to D_116_ and the S_114_ of GhDi19-2 was mutated to D_114_. S, serine (Ser); D, aspartic acid (Asp).

To explore the functional relationship of GhDi19s and CDPK, loss-of-function mutation of CPK11 (*Atcpk11-2*, SALK_054495) was employed in this study. A single copy of T-DNA was inserted into the genome of the *Atcpk11-2* mutant, generating a 39-bp deletion from 320 to 358 bp downstream of the translation start codon^11^. Quantitative RT-PCR analysis demonstrated that *AtCPK11* mRNA was undetectable in *Atcpk11-2* mutant. Meanwhile, S_116_ was mutated to D_116_ in GhDi19-1 and S_114_ was mutated to D_114_ in GhDi19-2 for generating the constitutively activated GhDi19-1 and GhDi19-2 [named GhDi19-1(S116D) and GhDi19-2(S114D), respectively]. The coding sequences of *GhDi19-1*, *GhDi19-2*, *GhDi19-1(S116D)* and *GhDi19-2(S114D)* under the control of CaMV 35S promoter were introduced into *Atcpk11-2* mutant. Homozygotes of T3 generation of these GhDi19 transgenic lines in *Atcpk11-2* background were analyzed by quantitative RT-PCR to confirm the transgene expression (Fig. 3b). Seeds of wild type, *Atcpk11-2* mutant and GhDi19-1, GhDi19-2, GhDi19-1(S116D) and GhDi19-2(S114D) overexpression transgenic lines in *Atcpk11-2* background (*GhDi19-1*/*cpk11*, *GhDi19-2/cpk11*, *GhDi19-1(S116D)*/*cpk11* and *GhDi19-2(S114D)*/*cpk11*) were sowed on MS medium with or without ABA and NaCl, respectively. Seed germination rate of wild type and the transgenic lines was monitored under different abiotic stresses. On MS medium, all the seeds from wild type and the transgenic lines were able to fully germinate (≤4 days) after sowing, and there was no significant difference in seed germination rate between the transgenic lines and wild type. However, in the presence of 0.8 μM ABA, seeds of *Atcpk11-2* mutant germinated much earlier and faster than those of wild type, and the germination rate of *GhDi19-1/cpk11* and *GhDi19-2/cpk11* seeds was similar to wild type, whereas seeds of *GhDi19-1(S116D)/cpk11* and *GhDi19-2(S114D)/cpk11* germinated much later than wild type. After 4 days, about 60% of wild type, *GhDi19-1*/*cpk11* and *GhDi19-2/cpk11* seeds germinated, and approximately 75% of *Atcpk11-2* mutant seeds germinated, but only about 35% of *GhDi19-1(S116D)*/*cpk11* and *GhDi19-2(S114D)*/*cpk11* seeds germinated. After 6 days, seed germination rate of *Atcpk11-2* mutant reached to about 90%, but those of *GhDi19-1(S116D)*/*cpk11* and *GhDi19-2(S114D)*/*cpk11* transgenic lines reached to only approximately 40–50%. In the presence of 150 mM NaCl, seed germination rate of *Atcpk11-2* mutant was significantly higher than that of wild type. On the contrary, germination of the *GhDi19-1(S116D)*/*cpk11* and *GhDi19-2(S114D)*/*cpk11* transgenic seeds was inhibited much more by NaCl, compared with wild type. Approximately 80% of *Atcpk11-2* mutant seeds and 70% of wild type, *GhDi19-1/cpk11* and *GhDi19-2/cpk11* seeds, but only 50% of *GhDi19-1(S116D)/cpk11* and *GhDi19-2(S114D)/cpk11* transgenic seeds germinated after 4 days (Fig. 7a).

During early seedling development, no significant difference was observed among the transgenic variants and wild type when seedlings grew on MS medium. However, the seedlings of *Atcpk11-2* mutant grew better than those of wild type on ABA- and NaCl-containing medium, but the *GhDi19-1(S116D)*/*cpk11* and *GhDi19-2(S114D)*/*cpk11* transgenic seedlings were more sensitive to ABA and salt than wild type, while growth status of *GhDi19-1*/*cpk11* and *GhDi19-2/cpk11* transgenic seedlings was similar to wild type under ABA and NaCl treatments (Fig. 7b). Under ABA and NaCl treatments, cotyledon greening rate of *Atcpk11-2* mutant was significantly higher than that of wild type, while cotyledon greening rate of *GhDi19-1/cpk11* and *GhDi19-2/cpk11* transgenic lines was similar to wild type. In contrast, cotyledon greening of *GhDi19-1(S116D)/cpk11* and *GhDi19-2(S114D)/cpk11* transgenic lines was inhibited much more by ABA and NaCl, compared with wild type (Fig. 7c).

When the seedlings were transferred onto MS plates in the vertical position without any stress treatments, the root growth of all transgenic variants was almost as same as those of wild type. However, when the seedlings were transferred onto MS medium supplemented with 5 μM ABA or 150 mM NaCl for several days, primary root growth of *Atcpk11-2* mutant was much better than that of wild type, and root growth of *GhDi19-1*/*cpk11* and *GhDi19-2/cpk11* transgenic lines was similar to wild type, whereas root growth of the *GhDi19-1(S116D)*/*cpk11* and *GhDi19-2(S114D)*/*cpk11* transgenic seedlings was inhibited much more than that of wild type (Fig. 7d). Roots of *Atcpk11-2* mutant were longer than those of wild type, and root length of the *GhDi19-1/cpk11* and *GhDi19-2/cpk11* transgenic lines was similar to wild type, but roots of *GhDi19-1(S116D)/cpk11* and *GhDi19-2(S114D)/cpk11* transgenic seedlings were shorter than those of wild type under ABA and NaCl treatments (Fig. 7e). These results indicated that overexpression of GhDi19-1/-2 in *Atcpk11* background could recover the salt- and ABA-insensitive phenotype of the mutant, implying the other CDPK (besides CPK11) may also activate GhDi19-1/-2 in the transgenic *Arabidopsis* plants, and Ser phosphorylation is important for functionally activating GhDi19-1/-2 in response to salt stress and ABA signaling during seed germination and early seedling development.

## Discussion

A large number of genes encoding receptors, kinases, transcription factors and other signal molecules in plants are induced after exposure to various abiotic stresses^35^,^36^. These genes function in various ways to confer plants stress tolerance^37^,^38^,^39^. Due to much low similarity of the different Di19-related proteins outside the zinc finger domain, Di19 proteins may play diverse roles in plant development and in response to abiotic stresses^32^. Previous studies revealed that GhDi19-1 and GhDi19-2 are involved in plant response to salt stress and ABA signaling^34^. In this study, we further investigated the mechanism of GhDi19-1 and GhDi19-2 involving in response to salt stress and ABA signaling, and found the Ser site is essential for GhDi19-1 and GhDi19-2 proteins involving in plant response to salt and ABA during seed germination and early seedling development.

Modification of phosphorylation by kinases and of dephosphorylation by phosphatases is an important physiological process as plants respond to abiotic stresses and ABA signaling. Some ABA/stress signaling regulators are modulated at the post-translational level by changing their phosphorylation states^40^,^41^,^42^,^43^,^44^. Calcium-dependent protein kinases (CDPKs) are unique serine/threonine kinases in plants responding in abiotic stresses^45^. Their multifunctionality and signaling specificity may be conferred by their ability to phosphorylate different substrates. *Arabidopsis* CPK4 and CPK11 positively regulate ABA signaling via phosphorylating downstream ABA-responsive transcription factors in plants^11^. Furthermore, previous studies revealed that most of seven *Arabidopsis* AtDi19s are phosphorylated by CPK3 and CPK11 *in vitro* in a Ca^+^^2^-dependent manner^33^. Likewise, both GhDi19-1 and GhDi19-2 contain a conserved nuclear localization signal (NLS), in which two putative kinase phosphorylation sites (Thr114 and Ser116 in GhDi19-1, and Thr112 and Ser114 in GhDi19-2, respectively) are located^34^. In this study, our data indicated that GhDi19-1/-2 could be phosphorylated *in vitro* by CPK11 in a Ca^2+^-dependent manner, and the Ser site of GhDi19-1/-2 is required for phosphorylation and normal subcellular localization of GhDi19-1 and GhDi19-2 proteins. Previous study reported that CPK11 phosphorylates Ser104 and Ser107 sites within the AtDi19-1 bipartite NLS probably, but effects of Ser phosphorylation at different sites on Di19 proteins involving in plant response to salt and ABA are unknown so far^33^. In this study, our data demonstrated the Ser site (but not Thr site) is crucial for functionally activation of cotton Di19-1/-2 in response to salt stress and ABA signaling.

AtCPK11 interacts with AtDi19-1 protein in the cell nucleus, and increases AtDi19-1 transactivation of *PR1*, *PR2*, and *PR5* expression^31^. Furthermore, AtCPK4 and AtCPK11 overexpression transgenic plants display the hypersensitivity to ABA during seeds germination and seedlings growth^11^. Similar phenotype was also observed in the *GhDi19-1/-2* overexpression transgenic *Arabidopsis*^34^. These findings indicated that there is a specific functional relationship of cotton Di19s and CDPKs. Additionally, overexpression of GhDi19-1/-2 in *Atcpk11* could recover the salt- and ABA-insensitive phenotype of the mutant, implying the other kinases (besides CPK11) may also activate GhDi19-1/-2 in the transgenic *Arabidopsis* plants.

Based on the data presented in this study, we thought that Di19 proteins may be potential downstream targets of CDPKs in ABA signal pathway during early plant growth and development. When plants are exposed to abiotic stress stimuli (such as drought and high salinity etc.), concentrations of intracellular ABA and Ca^2+^ may be increased in plants for responding the environmental stress signaling. Subsequently, Ser/Thr kinases (e.g. CDPK) are activated by ABA and in turn the activated kinases phosphorylate Di19 proteins at Ser site in the cell nucleus. Finally, the activated Di19 proteins transduce the signals to downstream ABA- and stress-responsive genes, thereby promoting plants response to abiotic stresses.

## Methods

### Plants materials and growth conditions

A T-DNA insertion mutant (named *Atcpk11-2*, SALK_054495) of *Arabidopsis* CPK11 (AT3g05700) was obtained from ABRC (www.arabidopsis.org/abrc). Seeds of *Arabidopsis thaliana* (*Columbia* ecotype), including wild type, mutant and transgenic lines, were surface-sterilized with 75% ethanol for 1 min and 10% NaClO for 5 min, followed by washing with sterile water. The sterilized *Arabidopsis* seeds were plated on Murashige and Skoog (MS) medium. After stratification at 4 °C for 2 days, the plates were transferred to a plant growth incubator (Sanyo, Osaka, Japan) for seed germination (16 h light/8 h dark at 22 °C) ten days later, seedlings were transplanted in soil and grown in a growth room under the conditions of 16 h light/8 h dark cycle at 22–24 °C. Tissues were derived from these seedlings for RNA extraction.

### Generation of transgenic plant and mutant lines

The coding sequences of cotton *GhDi19-1* and *GhDi19-2* genes, amplified from its cDNA by PCR with the proofreading *pfu* DNA polymerase, were cloned into pBI121 vector with *BamH* I*/Sac* I sites to replace the *GUS* gene, respectively^34^.

To generate loss-of-phosphorylation *GhDi19-1* mutants [GhDi19-1(S116A), GhDi19-1(T114A) and GhDi19-1(S/T-A/A)] and *GhDi19-2* mutants [GhDi19-2(S114A), GhDi19-2(T112A) and GhDi19-2(S/T-A/A)], primer-based site-directed mutations were performed, using primer pairs as follows: *GhDi19-1(S116A)* 5′-TCCTCAGAAGAGCAAGCGTGGAATGAG-3′ and 5′-CTCATTCCACGCTTGCTCTTCTGAGGA-3′; *GhDi19-1(T114A)* 5′-CAGAAGAGAAAGCGCGGAATGAGATCC-3′ and 5′-GGATCTCATTCCGCGCTTTCTCTTCTG-3′; *GhDi19-1(S/T-A/A)* 5′-CCTCAGAAGAGCAAGCGCGGAATGA-3′ and 5′-TCATTCCGCGCTTGCTCTTCTGAGG-3′; *GhDi19-2(S114A)* 5′-GAAGAGAAAGCGCTGAATGAGATCCAC-3′ and 5′-GTGGATCTCATTCAGCGCTTTCTCTTC-3′; *GhDi19-2(T112A)* 5′-CTTTCCTCAGAAGAGCAAGCGTTGAATGAG-3′ and 5′-CTCATTCAACGCTTGCTCTTCTGAGGAAAG-3′; *GhDi19-2(S/T-A/A)* 5′-CCTCAGAAGAGCAAGCGCTGAATGAGATC-3′ and 5′-GATCTCATTCAGCGCTTGCTCTTCTGAGG-3′. Mutants were introduced by QuickChange site-directed mutagenesis (Stratagene) and confirmed by sequencing. The coding sequences of *GhDi19-1(S116A)*, *GhDi19-1(T114A)*, *GhDi19-1(S/T-A/A)* and *GhDi19-2(S114A)*, *GhDi19-2(T112A)*, and *GhDi19-2(S/T-A/A)* were cloned into pBI121 vector, respectively.

To introduce the Ser116-to-Asp (S116D) mutation of GhDi19-1 or the Ser114-to-Asp (S114D) mutation of GhDi19-2 for constitutively activated GhDi19-1 or GhDi19-2, primer-based site-directed mutations were performed, using primers as follows: *GhDi19-1(S116D)* 5′-CTCATTCCGACCTTGACCTTCTGAGGA-3′ and 5′- TCCTCAGAAGGTCAAGGTCGGAATGAG-3′; *GhDi19-2(S114D)* 5′-CATTCAGACCTTGACCTTCTGAGGAAAG-3′ and 5′-CTTTCCTCAGAAGGTCAAGGTCTGAATG-3′. The coding sequences of *GhDi19-1(S-D)* and *GhDi19-2(S-D)* were cloned into pCAMBIA1301 vector. Mutant alleles of *Atcpk11-2* (SALK_054495) were used for experiments^11^. Complementation experiments using *GhDi19-1/-2* and *GhDi19-1/-2(S-D)* transgenes were done in *Atcpk11-2* background.

All generated binary vectors were transformed into *Agrobacterium turmefaciens* strain GV3101. *Arabidopsis* transformation was performed by the floral dip method^46^, and transformants were identified on selective medium with kanamycin or hygromycin.

### Quantitative RT-PCR analysis

Total RNA was extracted from 10-day-old *Arabidopsis* seedlings of wild type, *GhDi19-1-*, *GhDi19-1(S116A)*-, *GhDi19-1(T114A)*- and *GhDi19-1(S/T-A/A)*-overexpression transgenic lines, *GhDi19-2-*, *GhDi19-2(S114A)-*, *GhDi19-2(T112A)-* and *GhDi19-2(S/T-A/A)*-overexpression transgenic lines, *Atcpk11* mutant, *GhDi19-1(S-D)*- and *GhDi19-2(S-D)*-overexpression transgenic lines in *Atcpk11*-2 mutant. Real-time quantitative RT-PCR (qRT-PCR) analysis was performed as described as previously^47^. Primer pairs for qRT-PCR analysis are as follows: *GhDi19-1* 5′-ATGGATGCTGATTCATGGAGT-3′ and 5′-TTATAAAATTTCATCAGGCAT-3′; *GhDi19-1(S116A)* 5′-ATGGATGCTGATTCATGGAGT-3′ and 5′-CTCATTCCACGCTTGCTCTTCTGAGGA-3′; *GhDi19-1(T114A)* 5′-ATGGATGCTGATTCATGGAGT-3′ and 5′-GGATCTCATTCCGCGCTTTCTCTTCTG-3′; *GhDi19-1(S/T-A/A)* 5′-ATGGATGCTGATTCATGGAGT-3′ and 5′-TCATTCCGCGCTTGCTCTTCTGAGG-3′; *GhDi19-1(S116D)* 5′-ATGGATGCTGATTCATGGAGT-3′ and 5′-CTCATTCCGACCTTGACCTTCTGAGGA-3′; *GhDi19-2* 5′-ATGGATGCTGATCCATGGAC-3′ and 5′-TCATAAAACATCATCAAGAAT-3′; *GhDi19-2(S114A)* 5′-ATGGATGCTGATCCATGGAC-3′ and 5′-CTCATTCAACGCTTGCTCTTCTGAGGAAAG-3′; *GhDi19-2(T112A)* 5′-ATGGATGCTGATTCATGGAGT-3′ and 5′-GTGGATCTCATTCAGCGCTTTCTCTTC-3′; *GhDi19-2(S/T-A/A)* 5′-ATGGATGCTGATTCATGGAGT-3′ and 5′-GATCTCATTCAGCGCTTGCTCTTCTGAGG-3′; *GhDi19-2(S114D)* 5′-ATGGATGCTGATCCATGGAC-3′ and 5′-CATTCAGACCTTGACCTTCTGAGGAAAG-3′; *Atcpk11-2* 5′- GAGAGAGTCAAAAAAATTGGAGAA-3′ and 5′-AAACCAATTAGGCGATGAACC-3′. Expression level of *Arabidopsis ACTIN2* was monitored with forward 5′-GAAATCACAGCACTTGCACC-3′ and reverse 5′-AAGCCTTTGATCTTGAGA GC-3′ primers to serve as an internal control. For all the above qRT-PCR reactions, the assays were repeated three times along with three independent repetitions of the biological experiments, and means of three biological experiments were calculated for estimating gene expression levels.

### Phenotypic analysis of the transgenic *Arabidopsis* plants

Homozygous plants (T3 and T4 generations) of the *GhDi19-1/-2 *transgenic lines and their site-mutations were used for phenotypic analysis, employing wild type, mutant and the transgenic line harboring the “empty vector” (i.e. pBI121-eGFP) as controls. We first tested a series of ABA and NaCl concentrations in the pre-experiments, and determined which concentration of abscisic acid (ABA) or NaCl is suitable to the respective experiments. Then, seeds of wild type and independent transgenic lines, mutants, or transgenic mutants were germinated on MS medium supplemented without or with 150 mM NaCl, 0.8 and l μM ABA, respectively. The seeds were incubated at 4 °C for 2 days and then transferred into a plant growth incubator at 22 °C conditions (16 h light/8 h dark). Seeds were considered successfully germinated when radicles completely penetrated the seed coats. Germination rate and proportion of seedlings with opened green cotyledons were expressed as a percentage of the total number of seeds plated.

The seedling growth experiments were performed as described previously^11^. Seeds were germinated after stratification on MS medium for 48 h and then transferred onto MS medium containing 150 mM NaCl and 5 μM ABA in the vertical position. The growth status of seedling was recorded for 10 days and the length of seedling primary roots was measured at tenth day after the transfer. In addition, seedling growth was also assessed by directly planting the seeds on NaCl- or ABA-containing MS medium to investigate the response of seedling growth to salt or ABA for 10 days.

Statistical analysis was performed in all the experiments. At least 300 seeds of each line were used for analyzing seed germination rate and cotyledon greening rate, and 30–60 seedlings in vertical cultivation were used for measuring root length. The experiments were repeated at least three times with three technical replications.

### Subcellular localization of GhDi19-1 and GhDi19-1 protein

The coding sequences of *GhDi19-1*, *GhDi19-1(S116D)*, *GhDi19-1(S116A)*, *GhDi19-1(T114A)*, *GhDi19-1(S/T-A/A)*, *GhDi19-2*, *GhDi19-2(S114D)*, *GhDi19-2(S114A)*, *GhDi19-2(T112A)* and *GhDi19-2(S/T-A/A)* were cloned into pBI121-*eGFP* vector to generate GhDi19-1:eGFP, GhDi19-1(S116D):eGFP, GhDi19-1(S116A):eGFP, GhDi19-1(T114A):eGFP, GhDi19-1(S/T-A/A):eGFP, GhDi19-2:eGFP, GhDi19-2(S114D):eGFP, GhDi19-2(S114A):eGFP, GhDi19-2(T112A):eGFP and GhDi19-2(S/T-A/A):eGFP construct, respectively, and then introduced into *Arabidopsis* by the floral dip method. The harvested seeds were germinated on selective MS medium for selecting transgenic plants. Subsequently, GFP fluorescence in root cells of the transgenic seedlings was observed under a SP5 Meta confocal laser microscope (Leica, Germany) with a filter set (488 nm for excitation and 506 ∼ 538 nm for emission). SP5 software (Leica, Germany) was employed to record and process the digital images taken. Primers used in vector construction are as follows: *GhDi19-1* 5′-CTTGGATCCATGGATGCTGATTCATGGAG-3′ and 5′-GGGTCTAGATTATAAAATTTCATCAGGC-3′; *GhDi19-2* 5′-CTTGGATCCATGGATGCTGATCCATGGAC-3′ and 5′-GGGTCTAGATCATAAAACATCATCAAGAA-3′; *GhDi19-1(S116D)* 5′-CTCATTCCGACCTTGACCTTCTGAGGA-3′ and 5′-TCCTCAGAAGGTCAAGGTCGGAATGAG-3′; *GhDi19-2(S114D)* 5′-CATTCAGACCTTGACCTTCTGAGGAAAG-3′ and 5′-CTTTCCTCAGAAGGTCAAGGTCTGAATG-3′; *GhDi19-1(S116A)* 5′-TCCTCAGAAGAGCAAGCGTGGAATGAG-3′ and 5′-CTCATTCCACGCTTGCTCTTCTGAGGA-3′; *GhDi19-2(S114A)* 5′-GAAGAGAAAGCGCTGAATGAGATCCAC-3′ and 5′-GTGGATCTCATTCAGCGCTTTCTCTTC-3′; *GhDi19-1(T114A)* 5′-CAGAAGAGAAAGCGCGGAATGAGATCC-3′ and 5′-GGATCTCATTCCGCGCTTTCTCTTCTG-3′; *GhDi19-2(T112A)* 5′-CTTTCCTCAGAAGAGCAAGCGTTGAATGAG-3′ and 5′-CTCATTCAACGCTTGCTCTTCTGAGGAAAG-3′; *GhDi19-1(S/T-A/A)* 5′-CCTCAGAAGAGCAAGCGCGGAATGA-3′ and 5′-TCATTCCGCGCTTGCTCTTCTGAGG-3′; *GhDi19-2(S/T-A/A)* 5′-CCTCAGAAGAGCAAGCGCTGAATGAGATC-3′ and 5′-GATCTCATTCAGCGCTTGCTCTTCTGAGG-3′.

### *In vitro* phosphorylation assay

The coding sequences of *AtCPK11*, *GhDi19-1*, *GhDi19-1(S116A)*, *GhDi19-2* and *GhDi19-2(S114A)* were inserted downstream the *malE* gene, which encodes maltose-binding protein (MBP), in pMAL-c2X vector for expressing MBP-AtCPK11, MBP-GhDi19-1, MBP-GhDi19-1(S116A), MBP-GhDi19-2 and MBP-GhDi19-2(S114A) fusion proteins, respectively. *In vitro* phosphorylation assay was performed for the fusion proteins and MBP protein (control) purified from *Escherichia coli* strain BL21 by MBP′s affinity for maltose (NEW ENGLAND), according to the method described previously^48^,^49^. Primers used in vector construction are as follows: *AtCPK11* 5′-CTTGGATCCATGGAGACGAAGCCAAACCCTAG-3′ and 5′-GGGGTCGACTCAGTCATCAGATTTTTCACCA-3′; *GhDi19-1* 5′-CTTGGATCCATGGATGCTGATTCATGGAG-3′ and 5′-GGGTCTAGATTATAAAATTTCATCAGGC-3′; *GhDi19-2* 5′-CTTGGATCCATGGATGCTGATCCATGGAC-3′ and 5′-GGGTCTAGATCATAAAACATCATCAAGAA-3′; *GhDi19-1(S116A)* 5′-CTCATTCCACGCTTGCTCTTCTGAGGA-3′ and 5′-TCCTCAGAAGAGCAAGCGTGGAATGAG-3′; *GhDi19-2(S114A)* 5′-CTCATTCAACGCTTGCTCTTCTGAGGAAAG-3′ and 5′-CTCATTCAACGCTTGCTCTTCTGAGGAAAG-3′.

## Additional Information

**How to cite this article**: Qin, L.-X. *et al.* Phosphorylation of serine residue modulates cotton Di19-1 and Di19-2 activities for responding to high salinity stress and abscisic acid signaling. *Sci. Rep.* **6**, 20371; doi: 10.1038/srep20371 (2016).

## References

[b1] Xiong L. M., Schumaker K. S. & Zhu J. K. Cell signaling during cold, drought, and salt stress. Plant Cell 14, 165–183 (2002). 12045276 10.1105/tpc.000596 PMC151254

[b2] Jakab G. *et al.* Enhancing *Arabidopsis* salt and drought stress tolerance by chemical priming for its abscisic acid responses. Plant Physiol. 139, 267–274 (2005). 16113213 10.1104/pp.105.065698 PMC1203376

[b3] Shinozaki K., Yamaguchi-Shinozaki K. & Seki M. Regulatory network of gene expression in the drought and cold stress responses. Curr. Opin. Plant Biol. 6, 410–417 (2003). 12972040 10.1016/s1369-5266(03)00092-x

[b4] Huang J. *et al.* A TFIIIA-type zinc finger protein confers multiple abiotic stress tolerances in transgenic rice (*Oryza sativa* L.). Plant Mol. Biol. 80, 337–350 (2012). 22930448 10.1007/s11103-012-9955-5

[b5] Belin C., Megies C., Hauserová E. & Lopez-Molina L. Abscisic acid represses growth of the *Arabidopsis* embryonic axis after germination by enhancing auxin signaling. Plant Cell 21, 2253–2268 (2009). 19666738 10.1105/tpc.109.067702 PMC2751952

[b6] Jiang M. & Zhang J. Cross-talk between calcium and reactive oxygen species originated from NADPH oxidase in abscisic acidinduced antioxidant defense in leaves of maize seedlings. Plant Cell Environ. 26, 929–939 (2003). 12803620 10.1046/j.1365-3040.2003.01025.x

[b7] Zhang A., Jiang M., Zhang J., Tan M. & Hu X. Mitogen-activated protein kinase is involved in abscisic acid-induced antioxidant defense and acts downstream of reactive oxygen species production in leaves of maize plants. Plant Physiol. 141, 475–487 (2006). 16531486 10.1104/pp.105.075416 PMC1475456

[b8] Xing Y., Jia W. & Zhang J. AtMKK1 mediates ABA-induced *CAT1* expression and H_2_O_2_ production via AtMPK6-coupled signaling in *Arabidopsis*. Plant J. 54, 440–451 (2008). 18248592 10.1111/j.1365-313X.2008.03433.x

[b9] Shi B. *et al.* OsDMI3-mediated activation of OsMPK1 regulates the activities of antioxidant enzymes in abscisic acid signaling in rice. Plant Cell Environ. 37, 341–352 (2014). 23777258 10.1111/pce.12154

[b10] Ding Y. F. *et al.* ZmCPK11 is involved in abscisic acid-induced antioxidant defense and functions upstream of ZmMPK5 in the abscisic acid signaling in maize. J. Exp. Bot. 64, 871–884 (2013). 23268839 10.1093/jxb/ers366 PMC3580805

[b11] Zhu S. Y. *et al.* Two calcium-dependent protein kinases, CPK4 and CPK11, regulate abscisic acid signal transduction in *Arabidopsis*. Plant Cell 19, 3019–3036 (2007). 17921317 10.1105/tpc.107.050666 PMC2174700

[b12] Yamaguchi-Shinozaki K. & Shinozaki K. Transcriptional regulatory networks in cellular responses and tolerance to dehydration and cold stresses. Annu. Rev. Plant Biol. 57, 781–803 (2006). 16669782 10.1146/annurev.arplant.57.032905.105444

[b13] Nakashima K., Ito Y. & Yamaguchi-Shinozaki K. Transcriptional regulatory networks in response to abiotic stresses in *Arabidopsis* and grasses. Plant Physiol. 149, 88–95 (2009). 19126699 10.1104/pp.108.129791 PMC2613698

[b14] Kodaira K. S. *et al.* *Arabidopsis* Cys2/His2 zinc-finger proteins AZF1 and AZF2 negatively regulate abscisic acid repressive and auxin-inducible genes under abiotic stress conditions. Plant Physiol. 157, 742–756 (2011). 21852415 10.1104/pp.111.182683 PMC3192566

[b15] Lindemose S., O’Shea C., Jensen M. K. & Skriver K. Structure, function and networks of transcription factors involved in abiotic stress responses. Int. J. Mol. Sci. 14, 5842–5878 (2013). 23485989 10.3390/ijms14035842 PMC3634440

[b16] Zhang J. Z., Creelman R. A. & Zhu J. K. From laboratory to field: using information from *Arabidopsis* to engineer salt, cold, and drought tolerance in crops. Plant Physiol. 135, 615–621 (2004). 15173567 10.1104/pp.104.040295 PMC514097

[b17] Shinozaki K. & Yamaguchi-Shinozaki K. Gene networks involved in drought stress response and tolerance. J. Exp. Bot. 58, 221–227 (2007). 17075077 10.1093/jxb/erl164

[b18] Takatsuji H. Zinc-finger proteins, the classical zinc finger emerges in contemporary plant science. Plant Mol. Biol. 39, 1073–1078 (1999). 10380795 10.1023/a:1006184519697

[b19] Pabo C. O., Peisach E. & Grant R. A. Design and selection of novel Cys2His2 zinc finger proteins. Annu. Rev. Biochem. 70, 313–340 (2001). 11395410 10.1146/annurev.biochem.70.1.313

[b20] Wolfe S. A., Nekludova L. & Pabo C. O. DNA recognition by Cys2His2 zinc finger proteins. Annu. Rev. Biophys. Biomol. Struct. 29, 183–212 (2000). 10940247 10.1146/annurev.biophys.29.1.183

[b21] Agarwal P. *et al.* Genome-wide identification of C2H2 zinc-finger gene family in rice and their phylogeny and expression analysis. Plant Mol. Biol. 65, 467–485 (2007). 17610133 10.1007/s11103-007-9199-y

[b22] Ciftci-Yilmaz S. & Mittler R. The zinc finger network of plants. Cell Mol. Life Sci. 65, 1150–1160 (2008). 18193167 10.1007/s00018-007-7473-4 PMC11131624

[b23] Sakamoto H. *et al.* *Arabidopsis* Cys2/His2-type zinc-finger proteins function as transcription repressors under drought, cold and high-salinity stress conditions. Plant Physiol. 136, 2734–2746 (2004). 15333755 10.1104/pp.104.046599 PMC523337

[b24] Mittler R. *et al.* Gain- and loss-of-function mutations in Zat10 enhance the tolerance of plants to abiotic stress. FEBS Lett 580, 6537–6542 (2006). 17112521 10.1016/j.febslet.2006.11.002 PMC1773020

[b25] Xu S., Wang X. & Chen J. Zinc finger protein 1 (ThZF1) from salt cress (*Thellungiella halophila*) is a Cys-2/His-2-type transcription factor involved in drought and salt stress. Plant Cell Rep. 26, 497–506 (2007). 17024447 10.1007/s00299-006-0248-9

[b26] Xu D. Q. *et al.* Overexpression of a TFIIIA-type zinc finger protein gene ZFP252 enhances drought and salt tolerance in rice (*Oryza sativa* L.). FEBS Lett 582, 1037–1043 (2008). 18325341 10.1016/j.febslet.2008.02.052

[b27] Sun S. J. *et al.* Functional analysis of a novel Cys2/His2-type zinc finger protein involved in salt tolerance in rice. J. Exp. Bot. 61, 2807–2818 (2010). 20460361 10.1093/jxb/erq120 PMC2882275

[b28] Huang X. Y., Chao D. Y., Gao J. P., Zhu M. Z., Shi M. & Lin H. X. A previously unknown zinc finger protein, DST, regulates drought and salt tolerance in rice via stomatal aperture control. Genes Dev. 23, 1805–1817 (2009). 19651988 10.1101/gad.1812409 PMC2720257

[b29] Gosti F., Bertauche N., Vartanian N. & Giraudat J. Abscisic acid-dependent and-independent regulation of gene expression by progressive drought in *Arabidopsis thaliana*. Mol. Gen. Genet. 246, 10–18 (1995). 7823904 10.1007/BF00290128

[b30] Milla M. A. R., Townsend J., Chang I. F. & Cushman J. C. The *Arabidopsis* AtDi19 gene family encodes a novel type of Cys2/His2 zinc-finger protein implicated in ABA-independent dehydration, high-salinity stress and light signaling pathways. Plant Mol. Biol. 61, 13–30 (2006a). 16786289 10.1007/s11103-005-5798-7

[b31] Liu W. X., Zhang F. C., Zhang W. Z., Song L. F., Wu W. H. & Chen Y. F. *Arabidopsis* Di19 functions as a transcription factor and modulates PR1, PR2, and PR5 expression in response to drought stress. Mol. Plant 6, 1487–1502 (2013). 23404561 10.1093/mp/sst031

[b32] Qin L. X., Li Y., Li D. D., Xu W. L., Zheng Y. & Li X. B. *Arabidopsis* drought-induced protein Di19-3 participates in plant response to drought and high salinity stresses. Plant Mol. Biol. 86, 609–625 (2014). 25218132 10.1007/s11103-014-0251-4

[b33] Milla M. A. R. *et al.* A novel yeast two-hybrid approach to identify CDPK substrates: characterization of the interaction between CPK11 and AtDi19, a nuclear zinc finger protein. FEBS Lett. 580, 904–911 (2006b). 16438971 10.1016/j.febslet.2006.01.013

[b34] Li G. *et al.* Two cotton Cys2/His2-type zinc-finger proteins, GhDi19-1 and GhDi19-2, are involved in plant response to salt/drought stress and abscisic acid signaling. Plant Mol. Biol. 74, 437–452 (2010). 20852918 10.1007/s11103-010-9684-6

[b35] Seki M. *et al.* Monitoring the expression pattern of 1300 *Arabidopsis* genes under drought and cold stresses by using a full-length cDNA microarray. Plant Cell 13, 61–72 (2001). 11158529 10.1105/tpc.13.1.61 PMC102214

[b36] Matsui A. *et al.* *Arabidopsis* transcriptome analysis under drought, cold, high-salinity and ABA treatment conditions using a tiling array. Plant Cell Physiol. 49, 1135–1149 (2008). 18625610 10.1093/pcp/pcn101

[b37] Yamaguchi-Shinozaki K. & Shinozaki K. A novel cis-acting element in an Arabidopsis gene is involved in responsiveness to drought, low-temperature, or high-salt stress. Plant Cell 6, 251–264 (1994). 8148648 10.1105/tpc.6.2.251 PMC160431

[b38] Shinozaki K. & Yamaguchi-Shinozaki K. Molecular response to dehydration and low temperature: differences and cross-talk between two stress signaling pathways. Curr. Opin. Plant Biol. 3, 217–223 (2000). 10837265

[b39] Zhu J. K. Salt and drought stress signal transduction in plants. Annu. Rev. Plant Biol. 53, 247–273 (2002). 12221975 10.1146/annurev.arplant.53.091401.143329 PMC3128348

[b40] Choi H., Hong J., Ha J., Kang J. & Kim S. Y. ABFs, a family of ABA-responsive element binding factors. J. Biol. Chem. 275, 1723–1730 (2000). 10636868 10.1074/jbc.275.3.1723

[b41] Johnson R. R., Wagner R. L., Verhey S. D. & Walker-Simmons M. K. The abscisic acid-responsive kinase PKABA1 interacts with a seed-specific abscisic acid response element-binding factor, TaABF, and phosphorylates TaABF peptide sequences. Plant Physiol. 130, 837–846 (2002). 12376648 10.1104/pp.001354 PMC166610

[b42] Song C. P. *et al.* Role of an *Arabidopsis* AP2/EREBP-type transcriptional repressor in abscisic acid and drought stress responses. Plant Cell 17, 2384–2396 (2005). 15994908 10.1105/tpc.105.033043 PMC1182496

[b43] Furihata T. *et al.* Abscisic aciddependent multisite phosphorylation regulates the activity of a transcription activator AREB1. Proc. Natl. Acad. Sci. USA 103, 1988–1993 (2006). 16446457 10.1073/pnas.0505667103 PMC1413621

[b44] Yoshida R., Umezawa T., Muzogushi T., Takahashi S., Takahashi F. & Shinozaki K. The regulatory domain of SRK2E/OST1/SnRK2.6 interacts with ABI1 and integrates abscisic acid (ABA) and osmotic stress signals controlling stomatal closure in *Arabidopsis*. J. Biol. Chem. 281, 5310–5318 (2006). 16365038 10.1074/jbc.M509820200

[b45] Ma S. Y. & Wu W. H. AtCPK23 functions in *Arabidopsis* responses to drought and salt stresses. Plant Mol. Biol. 65, 511–518 (2007). 17541706 10.1007/s11103-007-9187-2

[b46] Clough S. J. & Bent A. F. Floral dip: a simplified method for *Agrobacterium* mediated transformation of *Arabidopsis thaliana*. Plant J. 16, 735–743 (1998). 10069079 10.1046/j.1365-313x.1998.00343.x

[b47] Li X. B., Fan X. P., Wang X. L., Cai L. & Yang W. C. The cotton *ACTIN1* gene is functionally expressed in fibers and participates in fiber elongation. Plant Cell 17, 859–875 (2005). 15722467 10.1105/tpc.104.029629 PMC1069704

[b48] Mao G. H., Meng X. Z., Liu Y. D., Zheng Z. Y., Chen Z. X. & Zhang S. Q. Phosphorylation of a WRKY transcription factor by two pathogen-responsive MAPKs drives phytoalexin biosynthesis in *Arabidopsis*. Plant Cell 23, 1639–1653 (2011). 21498677 10.1105/tpc.111.084996 PMC3101563

[b49] Chen L., Ren F., Zhou L., Wang Q. Q., Zhong H. & Li X. B. The *Brassica napus* Calcineurin B-Like 1/CBL-interacting protein kinase 6 (CBL1/CIPK6) component is involved in the plant response to abiotic stress and ABA signaling. J. Exp. Bot. 63, 6211–6222 (2012). 23105131 10.1093/jxb/ers273 PMC3481211

